# Evaluation of palatal dimensions in different facial patterns by using digital dental casts

**DOI:** 10.1590/2177-6709.27.5.e222115.oar

**Published:** 2022-11-28

**Authors:** Silvana Allegrini KAIRALLA, Leandro VELASCO, Andre Luis Lacerda BACHI, Lucia Hatsue YAMAMOTO, Mario CAPPELLETTE

**Affiliations:** 1 Universidade Federal de São Paulo (UNIFESP), Faculdade de Medicina, Departamento de Otorrinolaringologia - Cabeça e Pescoço, Disciplina de Otorrinolaringologia Pediátrica (São Paulo/SP, Brazil).; 2 Universidade Federal de São Paulo (UNIFESP), Departamento de Otorrinolaringologia - Cirurgia da Cabeça e do Pescoço, Laboratório de Pesquisa ORL (São Paulo/SP, Brazil).; 3 Universidade de Santo Amaro, Programa de Pós-graduação em Ciências da Saúde (São Paulo/SP, Brazil).

**Keywords:** Dental arches, Anatomy, Hard palate, Orthodontics

## Abstract

**Objective:**

To analyze the variations of hard palate volume in adults with normal occlusion and different facial types and patterns, by using a three-dimensional analysis on digital casts.

**Methods:**

The dental casts of 70 Caucasian adults (28 men, 42 women), mean age of 16.4 years (SD 1.3 years), were scanned by using a tridimensional scanner (Delcam PowerSHAPE™, 2010, Birmingham, UK). Close points were selected in the gingival and cervical regions on the lingual surface of the maxillary teeth, to analyze palatal morphology. The facial patterns and types, and the measurements (width, length, height, volume) of the space on the hard palate were compared using analysis of covariance (ANCOVA), with age as the covariate, and sex as the independent variable. The significance level of 5% (*p* < 0.05) was adopted.

**Results:**

This study showed that the measurements of the width and length were similar among the mesofacial, dolichofacial and brachyfacial facial types, although the height and volume of the space on the hard palate were slightly smaller in dolichofacial individuals, and both Pattern I and Pattern II individuals showed no significant changes for the four measurements. The mean values among facial patterns were: Pattern I - width 38.31±2.59 mm; length 37.44±2.42 mm; height 17.03±2.42 mm and volume 10.52±1.72 mm^3^; Pattern II - width 37.48±2.44 mm; length 37.48±2.44 mm; height 16.79±2.42 mm and volume 10.41±1.65 mm^3^ (*p*>0.05 for all variables).

**Conclusion:**

There were no significant differences for the facial patterns and facial types of the individuals compared in the analyzed sample.

## RESUMO

**Objetivo:**

Analisar as variações do volume do palato duro em adultos com oclusão normal e diferentes tipos e padrões faciais, por meio de análise tridimensional em modelos digitais.

**Métodos:**

Os modelos das arcadas dentárias de 70 adultos caucasianos (28 homens, 42 mulheres), média de idade de 16,4 anos (DP 1,3 anos), foram digitalizados usando um *scanner* tridimensional (Delcam PowerSHAPE™*,* 2010, Birmingham, Reino Unido). Pontos próximos foram selecionados nas regiões gengival e cervical na superfície lingual dos dentes superiores, para analisar a morfologia palatina. Os padrões e tipos faciais e as medidas (largura, comprimento, altura, volume) do espaço no palato duro foram comparados por meio de análise de covariância (ANCOVA), com a idade como covariável e o sexo como variável independente, a um nível de significância de 5% (*p*< 0,05).

**Resultados:**

O presente estudo mostrou que as medidas de largura e comprimento foram semelhantes entre os tipos faciais mesofacial, dolicofacial e braquifacial, embora a altura e o volume do espaço no palato duro tenham sido ligeiramente menores nos indivíduos dolicofaciais, e tanto os indivíduos do Padrão I quanto do Padrão II não apresentaram alterações significativas para as quatro medidas. Os valores médios entre os padrões faciais foram: Padrão I - largura 38,31±2,59 mm, comprimento 37,44±2,42 mm, altura 17,03±2,42 mm, volume 10,52±1,72 mm^3^; Padrão II - largura 37,48±2,44 mm, comprimento 37,48±2,44 mm, altura 16,79±2,42 mm, volume 10,41±1,65 mm^3^ (*p*>0,05 para todas as variáveis).

**Conclusão:**

Não houve diferenças significativas para os padrões faciais e tipos faciais dos indivíduos comparados na amostra analisada.

## INTRODUCTION

When examining an orthodontic technique, it is important to determine the mechanics and the consequences of its use, as well as its impact on the anatomical structures of the stomatognathic system. The lingual technique is extremely attractive from an aesthetic perspective, because it is considered “invisible” - as the brackets are positioned on the lingual surface of teeth. On the other hand, this technique is sometimes disregarded by potential users, due to several reasons, including: fear of tongue pain or of developing changes in speech, especially for patients in certain professions, such as speakers and those with artistic careers.

The first study describing the use of lingual brackets positioned on the lingual surface of teeth and a mushroom-shaped archwire for tooth correction was presented by Fujita^1^ in 1979. At the end of the 1970s, this technique was also being developed in the United States.^2^

The fact that the brackets were in direct contact with the tongue muscle drew the attention of professionals and patients, causing fear that the lingual appliance could cause pain. However, studies^3^ ^,^ ^4^ showed that adaptation to the lingual appliance is similar to that with the buccal orthodontic technique, and patient reports showed that some individuals could adapt more quickly than others, which may be related to the size of the dental arches, the position of the tongue and its relationship with the dental arches and, especially, the hard palate. Adaptation may also be related to the type of lingual bracket, how the lingual appliance is mounted, and the shape of the archwire used. Studies show that patients adapt to lingual appliances within three weeks,^3^ ^-^ ^5^ and the type of bracket used has a direct relationship with the patient’s adaptation to speech, comfort and painful symptoms. Furthermore, a higher bracket profile due to either the bracket’s metallic structure (bracket base) or its combination with resin to form a pad in individual bracket mountings, results in greater discomfort for the patient.^6^ ^,^ ^7^

The tongue is a muscle that is in direct contact with the hard palate and the lingual surface of teeth during swallowing and articulatory and chewing movements.^8^ Thus, some researchers have analyzed the width, length, height and volume of the hard palate^9^ because it has a close relationship with the tongue, not only during speech and chewing movements, but also during swallowing and resting, when the tongue remains supported on the structures of the hard palate and the lingual surface of the maxillary teeth for a long period of time.

Three-dimensional (3D) digitized models have enabled examination^10^ ^-^ ^12^ of the hard palate dimensions. However, other research tools are necessary to analyze the tongue volume.^13^ In addition, the hard palate, as part of the fixed scaffold of the skull, may be related to the facial patterns and facial types of individuals, which has led some researchers to investigate this correlation.^14^ ^-^ ^17^

Thus, the present study aimed to determine the width, length, height and volume of the space on the hard palate in a sample of dental cast, using resources such as a 3D scanner and the computer program Delcam PowerSHAPE™ 2010 (Birmingham, UK), in addition to verify the existence of a possible relationship between facial types and facial patterns.

## METHODS

The protocol of this observational analytical study was approved by the Research Ethics Committee of *Universidade Federal de São Paulo* (São Paulo/SP, Brazil) under the number 0388/2016. The sample consisted of 70 Brazilian Caucasian individuals (28 men and 42 women), with a minimum age of 15.0 years and a maximum age of 21.3 years (mean age 16.4 ± 1.3 years). The facial patterns and facial types of the patients were classified using lateral cephalometric radiographs and extraoral photographs of the smile in frontal and lateral views, and by subjective facial analysis.^18^ Among the sample, 43 patients were classified as having Pattern I face, and 27 patients had Pattern II face. The facial type analysis indicated 35 mesofacial, 28 brachyfacial and 7 dolichofacial patients.

The following inclusion criteria were used: individuals with normal natural occlusion, complete dental eruption, except for the third molars, and the presence of four occlusion keys.^19^ The four keys considered were a Class I molar relationship, crown angulation, crown inclination (considering the long axis of the teeth) and the curve of Spee (flat or smooth); rotations of up to 3 degrees and diastemas with spacing up to 0.5 mm were accepted. The exclusion criteria were the presence of odontogenic abnormalities, previous orthodontic treatment, and presence of the third molars.

**Figure 1:**
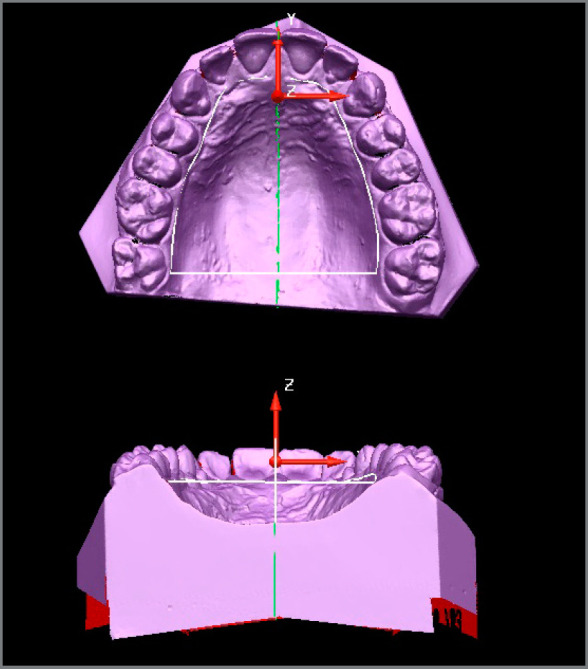
Model with the area delimited after joining the selected points.

The 70 cast models of the maxilla were scanned using a dw5-140 3D scanner (Dental Wings^®^, Montreal, Quebec, Canada) at the *Hospital da Face* (São Paulo/SP, Brazil). After obtaining the images of the scanned models, the images were read using Delcam PowerSHAPE™ software (2010, Birmingham, UK) (Fig 1).

**Figure 2:**
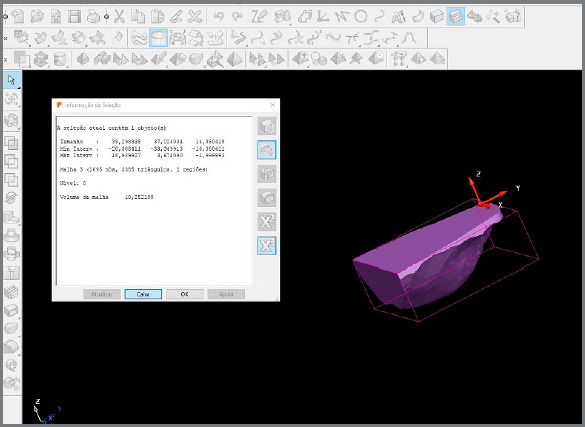
Area selected by the Delcam PowerSHAPE™ 2010 software.

**Figure 3:**
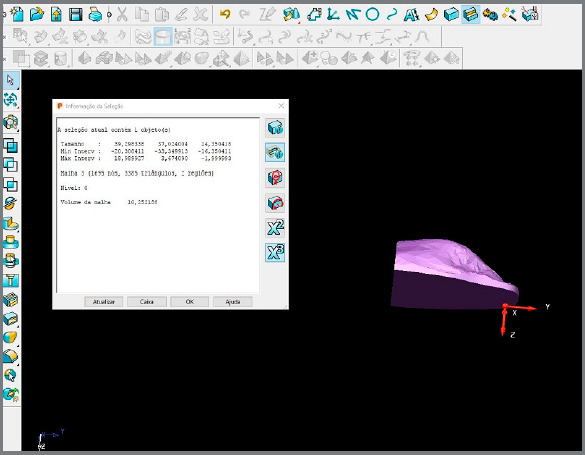
Lateral view of the area delimited by the Delcam PowerSHAPE™ 2010 software.

The *x-*, *y-* and *z-*axes were transferred to the maxillary model using a straight line starting at a point between the maxillary central incisors and ending near the distal surface of the second molars, passing exactly over the *y-*axis (Fig 2). Then, the models could be rotated on the computer screen such that the lingual surfaces could be observed in a frontal view, and the operator could define and locate exactly where the points should be placed. The points were placed using a tool in Delcam PowerSHAPE™ 2010 software (Fig 3).

The points were defined on the gingival surface approximately 1 mm from the cervical horizontal line of the gingiva on the lingual surface of the maxillary teeth, and from the second molar on the left side to the second molar on the right side. The location of the points were required to coincide with the continuation of the long axis of all maxillary anterior and posterior teeth.

After the points were defined, they were joined in the anterior and posterior regions, to delimit the space where the tongue could be positioned comfortably at rest on the structures of the hard palate (Fig 1). Delcam PowerSHAPE™ 2010 software tools were used to determine the width, length, height and volume of that space on the palate. First, the program selected and marked with a rectangle the area to be analyzed (Fig 2). Next, the program cut the selected area. This delimited area is shown in the Figure 3. Subsequently, these measurements were tabulated in EXCEL (Microsoft™, Redmond, Washington, USA) for statistical analysis.

## STATISTICAL ANALYSIS

Descriptive statistics, including means, standard deviations, and 95% confidence intervals (95% CI) were calculated for the measurements. The data were evaluated using IBM Statistical Package for Social Sciences software, version 24.0 for Windows (IBM Corp. Released 2016. IBM SPSS Statistics for Windows, Armonk, NY).

Based on the means and standard deviations obtained from the sample of 70 individuals, using a significance level of 5%, to verify a minimum difference of 10% between the groups, the sample had a power of 99% for width and length measurements, 81% for height and 73% for volume.

The intraclass correlation coefficient (ICC) was used for evaluating the intra-evaluator and inter-evaluator errors. The ICC (0.98; *p*< 0.0001) ensures the reliability of measurements by means of an evaluation of random error, by checking the consistency between the measurements. 

The normality of the data was evaluated using the Shapiro-Wilk test, whose results led to the conclusion that the variables studied had a normal distribution. To compare facial patterns (Table 1) and facial types (Table 2) with the width, length, height and volume of the space of the palate, analysis of covariance (ANCOVA) was used, with age as the covariate and sex as the independent variable. In all statistical tests, a significance level of 5% (*p*< 0.05) was adopted.^20^

**Table 1:** Comparison among facial patterns adjusted for sex and age, with respect to palate width, length, height and volume.

Pattern	Width		Length		Height		Volume	
	mean	SD	mean	SD	mean	SD	mean	SD
I (n = 43)	38.31	2.59	37.44	2.42	17.03	2.42	10.52	1.72
II (n = 27)	37.94	3.25	37.48	2.44	16.79	2.42	10.41	1.65
*p*	0.903 ns		0.757 ns		0.925 ns		0.834 ns	

SD = standard deviation, ns = not significant.

## RESULTS

The level of intraoberver and interobserver agreement was very strong (ICC > 0.98; *p*< 0.005). Mean values among facial patterns were: Pattern I - width 38.31 ± 2.59 mm, length 37.44±2.42 mm, height 17.03±2.42 mm, volume 10.52±1.72 mm^3^; Pattern II - width 37.48 ± 2.44 mm, length 37.48 ± 2.44 mm, height 16.79 ± 2.42 mm, volume 10.41±1.65 mm^3^ (*p*> 0.05 for all variables). 

Mean values among facial types were: Mesofacial - width 38.56±2.82 mm, length 37.72±2.47 mm, height 17.36±2.11 mm, volume 10.78 ± 1.75 mm^3^; Brachyfacial - width 37.81 ± 2.99 mm, length 37.00 ± 2.05 mm, height 16.60 ± 2.79 mm, volume 10.32 ± 1.60 mm^3^; Dolichofacial - width 37.61 ± 2.50 mm, length 37.93±3.42 mm, height 16.17±2.00 mm, volume 9.58±1.44 mm^3^ (*p*> 0.05).

## DISCUSSION

The 3D technology used in the present study is an effective tool^9^ ^-^ ^12^ ^,^ ^20^ and less invasive than cone beam computed tomography^21^, and it is probably more accurate than measurements of the transversal space of the hard palate in a linear manner, i.e., 2D measurements obtained based on the cusps of teeth.^14^ ^,^ ^22^ The morphology, size and shape of the palate were investigated in a longitudinal study spanning ten years in adult patients, using similar landmarks in the palate as those used in the present study.^9^ The results suggested that the shape of the hard palate does not seem to change in adult patients. The dimensions of the hard palate in children with different facial types also showed no significant difference in hard palate measurements among brachyfacial, mesofacial and dolichofacial children.^22^

Bracket bonding using the lingual technique can be performed in a simple (direct)^7^ or more complex (indirect)^23^ manner. The location of the bracket can be centered on the lingual surface or positioned more cervically.^23^ Bonding of the lingual brackets in a more cervical position is performed to reduce the existing step between the lingual surface (concave) of the canine and the lingual surface of the first premolar (convex), and in this case, a lingual straight wire is required. Although this strategy allows reduction of the typical insets and offsets of the lingual surfaces of the dental arches, which are concave and convex, unlike the flat vestibular surfaces, an individualized laboratory configuration is necessary. This configuration can sometimes hinder orthodontic mechanics in certain movements,^9^ ^,^ ^10^ ^,^ ^24^ ^,^ ^25^ and cause gingival inflammation, because the resin pads (the amount of composite resin) required to compensate for the distance between the lingual surface and the bracket base on these irregular surfaces^11^ ^,^ ^12^ ^,^ ^26^ ^,^ ^27^ will be greater in areas where more compensation is necessary. In these cases, the brackets may advance into the space occupied by the tongue, resulting in possible patient discomfort.^6^ Some brackets may be mounted with a resin pad, and others may be mounted without a pad. A bracket base close to the lingual surface will have a lower profile, and the appliance will invade less of the space occupied by the tongue. Therefore, the patient will have better adaptation in terms of speech and comfort.^5^

The results of this study showed that the space of the palate height (16.17 mm) and volume (9.58 mm^3^) were slightly smaller in dolichofacial individuals (Table 2); however, there were no significant differences. Another study^14^ showed a difference in the depth of the palate, with brachyfacial patients exhibiting a shallower palate than dolichofacial patients. Nevertheless, in the present sample with normal occlusion, the number of dolichofacial individuals was very small (n=7). But we can understand that malocclusion may play a role in facial architecture, especially during growth, for example, in the presence of deviations in the teeth eruption process or respiratory function. Children with mouth-breathing pattern showed a significant constriction of the maxillary arch, when compared to individuals with nasal breathing pattern with increased palatal height in the posterior region of the palate, especially at the level of the first permanent molars.^28^

**Table 2:** Comparison among facial types adjusted for sex and age, with respect to palate width, length, height and volume.

Types	Width		Length		Height		Volume	
	mean	SD	mean	SD	mean	SD	mean	SD
M (n = 35)	38.56	2.82	37.72	2.47	17.36	2.11	10.78	1.75
B (n = 28)	37.81	2.99	37.00	2.05	16.60	2.79	10.32	1.60
D (n = 7)	37.61	2.50	37.93	3.42	16.17	2.00	9.58	1.44
*p*	0.577 ns		0.424 ns		0.350 ns		0.215 ns	

SD = standard deviation, ns = not significant. M = mesofacial, D = dolichofacial and B = brachyfacial.

The subjective facial analysis is an efficient method, and there is an agreement between both subjective and cephalometric analyses in evaluations of the soft tissue.^15^ Regarding the skeletal pattern, the subjective method requires improvements in the morphological criteria used to discriminate the five facial patterns (Pattern I, Pattern II, Pattern III, Long Face and Short Face).^29^ This conclusion may have been derived from the opinions of other authors^30^ indicating that Patterns I and II are the most prevalent facial patterns, while the least prevalent one is the Short Face pattern. This information may explain the present findings, as only individuals with Patterns I and II were included. Thus, this result may have occurred because the individuals in the sample had normal occlusion; therefore, a balance is assumed to exist in the skeletal bases, with minimal discrepancies. Unfortunately, there is no scientific evidence to support this assumption. However, as observed in Table 1, the present study found that both Pattern I (width 38.31 mm, length 37.44 mm, height 17.03 mm, volume 10.52 mm^3^) and Pattern II (width 37.94 mm, length 37.48 mm, height 16.79 mm, volume 10.41mm^3^) individuals showed no significant changes in hard palate measurements.

Most studies using 3D technology to assess the dimensions of the hard palate were related to treatments for palatal disjunction^11,12,20,21^ and/or mouth-breathing in young patients,^31^ ^,^ ^32^ reinforcing that malocclusion may play a role in facial architecture. The present study used models from a sample of individuals with normal natural occlusion, and the results can serve as parameters for future research on biomechanics, configurations and lingual arch shapes,^8^ ^-^ ^12^ ^,^ ^23^ ^-^ ^27^ which are important factors to be considered for the patients’ adherence to the technique. Therefore, in the diagnosis of orthodontic treatment, the facial type, the stomatognathic functions, the musculature and occlusion are essential in clinical practice.^16^

Some considerations can be proposed, such as the need for further studies on the structure of the hard palate space, including the tongue muscle, to improve the reliability when correlating both structures within the skeletal scaffold.

## CONCLUSION

There were no correlations between facial Patterns I and II or the dolichofacial, mesofacial and brachyfacial facial types and the width, length, height or volume of space on the hard palate.
